# Molecular Detection and Phylogenetic Analysis of *Anaplasma* spp. in Ticks Collected from Grasslands near Livestock Farms in Gyeonggi and Gangwon Provinces, Republic of Korea

**DOI:** 10.3390/microorganisms13092008

**Published:** 2025-08-28

**Authors:** Hyeyeon Kim, Myungji Jo, Younhyoung Choi, Haeseung Lee, SoYoun Youn, Mi-Sun Yoo, Hyang-Sim Lee, Jae-Myung Kim, Kaifa Nazim, Eun Hee Yun, Min-Goo Seo, Sang-Joon Park, Man Hee Rhee, Seung-Hun Lee, SungShik Shin, Dongmi Kwak

**Affiliations:** 1 College of Veterinary Medicine, Kyungpook National University, Daegu 41566, Republic of Korea; sdcd33@knu.ac.kr (H.K.); jmj8161@knu.ac.kr (M.J.); goo4258@knu.ac.kr (Y.C.); koreasmg@knu.ac.kr (M.-G.S.); psj26@knu.ac.kr (S.-J.P.); rheemh@knu.ac.kr (M.H.R.); 2 Veterinary Epidemiology Division, Animal and Plant Quarantine Agency, Gimcheon 39660, Republic of Korea; lhspppp@knu.ac.kr; 3 Parasitic and Honeybee Disease Laboratory, Bacterial Disease Division, Department of Animal and Plant Health Research, Animal and Plant Quarantine Agency, Gimcheon 39660, Republic of Korea; syyoun@korea.kr (S.Y.); msyoo99@korea.kr (M.-S.Y.); leehs76@korea.kr (H.-S.L.); kimjm88@korea.kr (J.-M.K.); 4 Department of Veterinary Parasitology, Khalsa College of Veterinary & Animal Sciences, Punjab 143001, India; kaifa.nazim@gmail.com; 5 Department of Companion Animal Health, Yeungnam University College, Daegu 42415, Republic of Korea; yehpic@ync.ac.kr; 6 Institute for Veterinary Biomedical Science, College of Veterinary Medicine, Kyungpook National University, Daegu 41566, Republic of Korea; 7 College of Veterinary Medicine, Chungbuk National University, Cheongju 28644, Republic of Korea; dvmshlee@cbnu.ac.kr; 8 College of Veterinary Medicine, Chonnam National University, Gwangju 59626, Republic of Korea; sungshik@jnu.ac.kr

**Keywords:** *Anaplasma phagocytophilum*, tick, tick-borne pathogen, zoonosis, livestock farm

## Abstract

Ticks are hematophagous ectoparasites capable of transmitting a wide array of pathogens. The recent proliferation and geographic expansion of tick populations have intensified concerns regarding the escalating risk of tick-borne pathogen transmission. Among these, *Anaplasma phagocytophilum* poses a notable threat to both public and veterinary health due to its zoonotic potential. In this study, a total of 4316 ticks were collected from 16 pasture sites near livestock farms in Gyeonggi and Gangwon Provinces, Republic of Korea, between April and October 2024. Molecular epidemiological analysis was subsequently performed. Of the 400 tick pools tested, *Ixodes nipponensis* accounted for two *Anaplasma*-positive pools, *Haemaphysalis* spp. larvae for two pools, and *Haemaphysalis longicornis* for one pool. This study is the first to examine the infection rate of *A. phagocytophilum* in ticks collected from pasture sites near livestock farms in Gyeonggi and Gangwon Provinces. Although the observed infection rate was low, the presence of *A. phagocytophilum* in regions with potential human exposure emphasizes the risk of transmission. Importantly, the detection of *A. phagocytophilum* in larval-stage ticks suggests the possibility of transovarial transmission, meriting further investigation. These findings underscore the need for conducting surveillance and targeted preventive strategies to reduce the burden of tick-borne diseases in livestock-associated settings.

## 1. Introduction

Ticks are hematophagous ectoparasitic arthropods that attach to the skin of humans and animals to obtain a blood meal. They act as vectors for a broad array of pathogens, including viruses, bacteria, and protozoa, such as tick-borne encephalitis virus, *Anaplasma* spp., *Borrelia* spp., *Rickettsia* spp., and *Babesia* spp. [1,2].

Species of *Anaplasma* spp. have long been recognized as important pathogens in veterinary medicine, contributing to substantial economic losses in livestock production across both tropical and temperate regions for over a century [3]. The genus comprises several species, including *A. phagocytophilum*, *A. bovis*, and *A. platys*, which infect a wide range of hosts such as livestock, wildlife, and humans. Among these, *A. phagocytophilum* is of particular concern due to its zoonotic potential. This Gram-negative, obligate intracellular bacterium is transmitted primarily by ticks of the *Ixodes* genus and is capable of infecting numerous mammalian hosts, including ruminants, dogs, cats, and horses [4]. In humans, infection with *A. phagocytophilum* causes human granulocytic anaplasmosis (HGA), an acute febrile illness characterized by headache, myalgia, thrombocytopenia, and leukopenia [5]. Although most cases are self-limiting, severe complications and fatalities can occur if left untreated. Furthermore, asymptomatic infections are common, leading to underreporting and a likely underestimation of the true disease burden. Since its initial identification in the United States in 1994 [6], HGA has been reported in Europe in 1997 [7], China in 2009 [8], and Japan in 2013 [9]. In Republic of Korea, the first confirmed human case was documented in 2002 [10], with a notable increase in reported cases in subsequent years [11]. Although ticks remain the primary transmission vector, increasing evidence suggests that direct contact with the blood of infected animals may also contribute to human infection [12].

Strains of *A. phagocytophilum* that are pathogenic to humans are classified as the human-active variant, commonly referred to as *A. phagocytophilum* human-active (Ap-ha) [13]. In contrast, *A. phagocytophilum*-variant 1 (Ap-V1), identified in *Ixodes scapularis*, is considered non-pathogenic to humans and laboratory mice but has been reported to cause infection in goats, white-tailed deer, and ticks, suggesting host-specific pathogenicity [14].

In Republic of Korea, most studies investigating *A. phagocytophilum*-infected ticks in Gyeonggi (GG) and Gangwon (GW) Provinces have focused on ticks collected from humans [15,16], rodents [17], and dogs [18]. However, there has been a lack of research on ticks collected directly from pasturelands near livestock farms, despite the high likelihood of pathogen spillover from ticks to livestock and, ultimately, to humans. Notably, the GG and GW regions are susceptible to tick introduction through land-based routes from North Korea and China. Thus, research in these areas could provide indirect insights into cross-border pathogen transmission and the infection rate of tick-borne pathogens in North Korea, where direct surveillance remains infeasible. In light of the growing public health concern surrounding zoonotic tick-borne diseases, elucidating the infection rate and molecular characteristics of *A. phagocytophilum* in ticks from livestock-associated environments is essential for assessing transmission risk and informing the development of effective control strategies. Therefore, the objective of this study was to investigate the distribution, infection rate, and molecular characteristics of *Anaplasma* spp. in ticks collected from pastures surrounding livestock farms in the GG and GW Provinces. As the first study to examine *Anaplasma* spp. infection in ticks from these specific environments in Republic of Korea, the findings are expected to provide critical baseline data to support the enhancement of zoonotic disease surveillance systems.

## 2. Materials and Methods

### 2.1. Ethical Approval

Approval from the Institutional Animal Care and Use Committee of Kyungpook National University was not required for this study, conducted in 2024. The collection of questing ticks from the environment did not involve procedures that posed harm to animals. None of the collected ticks were from endangered species. Site-specific approvals were not necessary, as all collection sites were located outside livestock farms and protected areas.

### 2.2. Tick Collection and Species Identification

**Figure 1:**
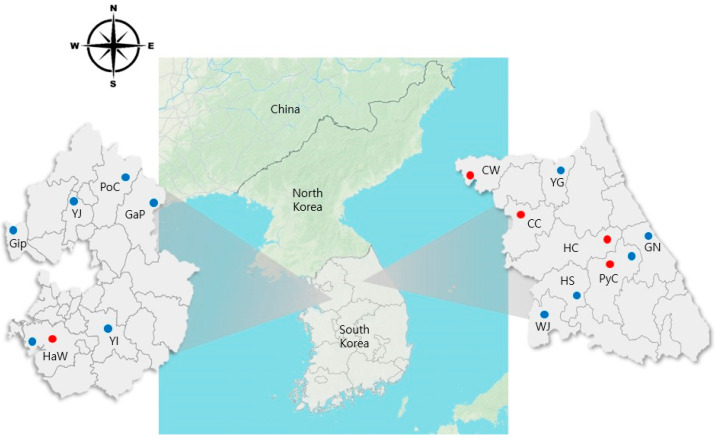
Map of tick collection sites in Gyeonggi [Hwaseong (HwS) and one site each in Gimpo (GiP), Yangju (YJ), Pocheon (PoC), Yongin (YI), and Gapyeong (GaP)] and Gangwon [Pyeongchang (PyC) and one site each in Hongcheon (HC), Gangneung (GN), Wonju (WJ), Cheorwon (CW), Chuncheon (CC), Hoengseong (HS), and Yanggu (YG)] Provinces (April–October, 2024). Blue circles indicate collection sites, whereas red circles represent sites where *Anaplasma* spp. positive samples were detected.

Ticks were collected monthly from April to October 2024 at 16 pasture sites located near livestock farms (cattle, deer, goat, and horse) in GG and GW Provinces, Republic of Korea. The collection was performed using flagging and dragging methods, which involved sweeping a flannel cloth across vegetation to obtain questing ticks at the square footage of the area with 20 × 20 m (Figure 1).

A total of 4316 ticks were submitted to the College of Veterinary Medicine at Kyungpook National University for analysis. Ticks were preserved in 70% ethanol and morphologically identified based on the reference [19]. Species identification, developmental stage, and sex were determined using a light microscope. The sexes of adult ticks were identified based on their dorsal scutum size. Adults and nymphs were identified to the species level, whereas larvae were classified to the genus level due to morphological similarities.

### 2.3. DNA Extraction and PCR Detection

Genomic DNA was extracted using the DNeasy Blood & Tissue Kit (Qiagen, Hilden, Germany). Ticks from each region were pooled by species and developmental stage, with a maximum of 59 larvae, 11 nymphs, or one adult per pool. Pooling was conducted after species identification to avoid cross-contamination of different species. Due to the morphological similarity of *Haemaphysalis* larvae, they were grouped as *Haemaphysalis* spp.

Polymerase Chain Reaction (PCR) amplification was conducted using the AccuPower HotStart PCR Premix Kit (Bioneer, Daejeon, Republic of Korea) on a Mastercycler nexus GSX1 thermal cycler (Eppendorf, Hamburg, Germany). To detect the 16S rRNA gene of *Anaplasma* spp., a nested PCR was performed using two primer sets: EE1 (5′ TCC TGG CTC AGA ACG AAC GCT GGC GGC 3′)/EE2 (5′ AGT CAC TGA CCC AAC CTT AAA TGG CTG 3′) to amplify nearly the full-length gene and EE3 (5′ GTC GAA CGG ATT ATT CTT TAT AGC TTG C 3′)/EE4 (5′ CCC TTC CGT TAA GAA GGA TCT AAT CTC C 3′) to amplify the V3–V8 hypervariable regions. This approach amplified a 924–926 bp region, as previously described [20]. The 1.5% agarose gel was run at 135 V for 30 min. Images were acquired using an ultraviolet transilluminator. A previously confirmed sample of *A. phagocytophilum* detected in cattle in Republic of Korea [21] served as a positive control, and a sample lacking a DNA template served as the negative control.

### 2.4. Sequencing and Phylogenetic Analyses

For direct DNA sequencing, all PCR-positive amplicons generated with the EE3/EE4 primer sets were subjected to Sanger sequencing by Macrogen (Daejeon, Republic of Korea). Sequencing alignment was performed using CLUSTAL Omega (v. 1.2.1, Bioweb, Ferndale, WA, USA), and the resulting sequences were compared with reference sequences from GenBank. Terminal regions were trimmed using MEGA11 [22] based on the sequences obtained in this study. Gaps and ambiguously aligned regions were excluded prior to phylogenetic analysis.

Phylogenetic analysis was performed using the maximum likelihood (ML) method in MEGA11. Genetic distances were computed with the Kimura 2-parameter model. For tree inference, the ML heuristic method was set to Nearest-Neighbor Interchange using the ML criterion, and the initial tree was automatically generated by the program.

### 2.5. Statistical Analysis

Statistical analyses were performed using the chi-square test in SPSS version 29.0 (SPSS Inc., Chicago, IL, USA). A *p*-value of <0.05 was considered statistically significant. The statistical analysis in this study was conducted to test the hypothesis that the infection rate of *Anaplasma* differs depending on tick species, sampling region, or developmental stage.

## 3. Results

### 3.1. Identification of Ticks

A total of 4316 ticks belonging to three genera and four species—*Haemaphysalis longicornis*, *Haemaphysalis flava*, *Ixodes nipponensis*, and *Amblyomma testudinarium*—were collected from 16 sites across GG (seven sites) and GW (nine sites) Provinces. Due to morphological similarity, larvae of *H. longicornis* and *H. flava* could not be reliably differentiated and were thus classified collectively as *Haemaphysalis* spp.

Of the total ticks collected, *H. longicornis* was the most prevalent species (*n* = 2488; 57.65%), followed by *Haemaphysalis* spp. (*n* = 1766; 40.91%), *H. flava* (*n* = 53; 1.23%), *I. nipponensis* (*n* = 8; 0.19%), and *A. testudinarium* (*n* = 1; 0.02%) (Table 1). *Haemaphysalis* spp. and *I. nipponensis* were identified in both GG and GW Provinces, whereas *A. testudinarium* was detected only once, in a specimen from the CC site in GW Province. This represents the first detection of *A. testudinarium* in GW Province, suggesting a previously undocumented regional distribution. Across both provinces, nymphs represented the most frequently encountered developmental stage (*n* = 2471; 57.25%; 1016 in GG and 1455 in GW), followed by larvae (*n* = 1766; 40.91%; 753 in GG and 1013 in GW), females (*n* = 58; 1.34%; 32 in GG and 26 in GW), and males (*n* = 21; 0.48%; thirteen in GG and eight in GW).

### 3.2. Infection Rate of Anaplasma spp. in Ticks

All 4316 ticks were grouped into 400 pools (171 pools from GG and 229 from GW), of which five pools (1.25%, 5/400) tested positive for *Anaplasma* spp. The estimated minimum infection rate (MIR) was 0.12%, with a 95% confidence interval (CI) of 0–10.1%. Sequencing confirmed the presence of *A. phagocytophilum* in all positive pools. Four of the five positive pools (1.75%, 4/229; MIR: 0.16%) originated from GW Province, whereas one (0.58%, 1/171; MIR: 0.06%) was from GG Province.

Tick abundance varied temporally throughout this study period. The highest total collection occurred in April (21.87%, 944/4316). Larvae were most frequently identified in October (38.67%, 683/1766), whereas nymphs were predominantly observed in April (37.07%, 916/2471). Among adults, female ticks were most abundant in July (53.45%, 31/58), whereas males were most commonly collected in both April and July (40.00%, 6/15 each). No larval ticks were collected in May, and adult females were absent in October. Adult males were not detected in September and October. These observations suggest limited larval and adult activity during these months, potentially influenced by seasonal environmental conditions. In contrast, nymphs were collected consistently throughout the study, indicating a relatively stable activity pattern.

As summarized in Table 2, positive pools were identified in ticks collected in April (MIR: 0.32%), August (MIR: 0.21%), and October (MIR: 0.15%), with the highest positivity rate observed among those collected in April.

By species and developmental stage, *A. phagocytophilum* was detected in two pools of *I. nipponensis* male (33.33%, 2/6; MIR: 33.33%), two pools of *Haemaphysalis* spp. larvae (3.77%, 2/53; MIR: 0.11%), and one pool of *H. longicornis* nymphs (0.40%, 1/249; MIR: 0.04%) (Table 3). No positive pools were observed for *H. flava* or *A. testudinarium*. The infection rate of *Anaplasma* differed significantly among tick species (chi-square test, *p* < 0.001).

Although a high MIR was observed for *I. nipponensis*, the small sample size could have led to an overestimation of the true infection rate. Due to the limited number of positive cases, the statistical significance of the calculated *p*-value should be interpreted with caution.

Regionally, the single positive pool in GG Province was identified in an adult male *I. nipponensis*. In GW Province, positive samples included one adult male *Ixodes*, one pool of *H. longicornis* nymphs, and two pools of *Haemaphysalis* larvae. The higher number of positive pools in GW Province compared to GG Province may reflect underlying ecological or climatic differences influencing the regional distribution of *Anaplasma* sp.

### 3.3. Molecular and Phylogenetic Analyses

Phylogenetic analysis demonstrated that all sequences obtained in this study were 100% identical to *A. phagocytophilum* sequences (OR287078, AF486636, MH338210, PP346249, or PP346263) available in the National Center for Biotechnology Information GenBank database. These reference sequences were selected based on the highest similarity (top hits) from BLAST (v. 2.17.0) searches using our representative sequences and included previously reported strains from various hosts and regions. Although the sequences showed 100% identity within the amplified 16S rRNA region, they were grouped into four distinct genotypes in the phylogenetic tree. Specifically, two identical sequences were derived from *I. nipponensis* pools, and three genotypes were identified among *Haemaphysalis* spp. pools.

**Figure 2:**
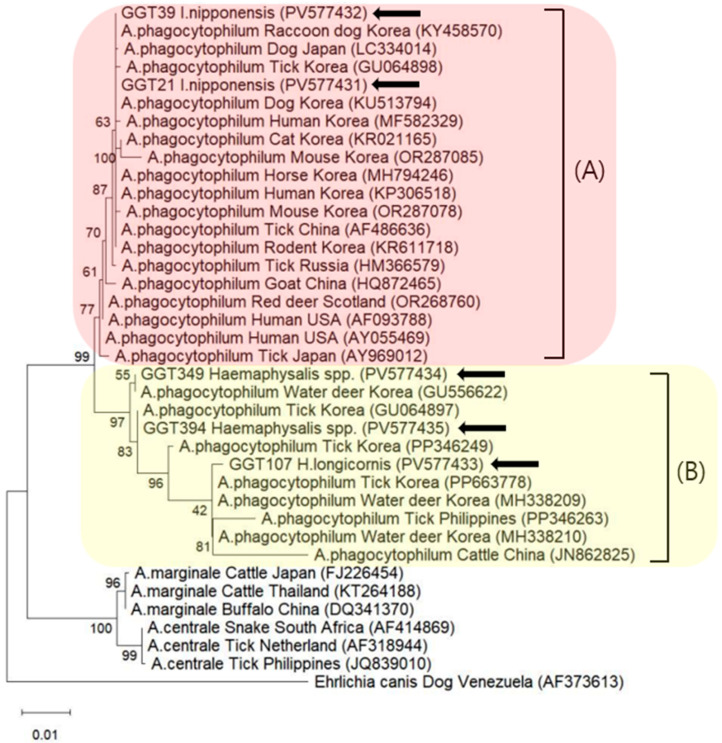
Phylogenetic tree constructed using the maximum likelihood method based on 16S rRNA sequences of *Anaplasma phagocytophilum*. Sequences analyzed in this study are indicated by black arrows. Clade A consisted of sequences known to be associated with human infection, whereas Clade B consisted of sequences that have not been implicated in human disease. Bootstrap analysis was performed with 5000 replicates.

Sequences from *Ixodes* ticks were grouped into Clade A, which exhibited high similarity to sequences known to be pathogenic to humans, whereas sequences from *Haemaphysalis* ticks were assigned to Clade B, which demonstrated low similarity to such pathogenic sequences (Figure 2).

All sequences obtained in this study were found to be 100% identical to *A. phagocytophilum*. The *A. phagocytophilum* sequence identified in *I. nipponensis* demonstrated phylogenetic similarity to OR287078, previously isolated from the Korean mouse, and AF486636, detected in Chinese *I. persulcatus*. The nucleotide sequence obtained from *H. longicornis* was identical to MH338210, originally isolated from a Korean roe deer, whereas the two sequences from the unclassified *Haemaphysalis* spp. matched PP346249 and PP346263, isolated from a Korean roe deer and a Philippine cattle tick, respectively. As only the 16S rRNA gene was employed for molecular analysis, the resolution for distinguishing between *Anaplasma* sp. may have been limited. To address this limitation, future studies should include experiments using alternative primers.

Representative sequences from this study were submitted to GenBank under accession numbers PV577431 to PV577435.

## 4. Discussion

In this study, four tick species (*H. longicornis*, *H. flava*, *I. nipponensis*, and *A. testudinarium*) were collected from grassland areas near livestock farms in GG and GW Provinces between April and October 2024. *H. longicornis* was the most prevalent species, accounting for 57.65% (2488/4316) of all collected specimens, followed by unidentified *Haemaphysalis* spp. (40.91%, 1766/4316), *H. flava* (1.23%, 53/4316), *I. nipponensis* (0.19%, 8/4316), and *A. testudinarium* (0.02%, 1/4316). Notably, only a single specimen of *A. testudinarium* was detected, exclusively in the northern region of GW Province. This species has previously been reported to inhabit predominantly southern regions of Republic of Korea, such as Gyeongnam and Jeonnam Provinces [23,24]. To our knowledge, this is the first report of *A. testudinarium* in GW Province, suggesting that climatic or environmental changes in this region may now support its habitat.

Additionally, the majority of ticks were collected in GW Province (57.97%, 2502/4316) compared to GG Province (42.03%, 1814/4316). This discrepancy may be explained by the higher number of collection sites in GW (nine sites) relative to GG (seven sites). To more accurately assess regional differences, future studies should standardize the number of collection sites across provinces.

Seasonal trends revealed that nymphal ticks were most prevalent from spring to midsummer, peaking in April. Larval activity increased from late summer to autumn, with the highest numbers recorded in October. Adult tick activity was highest in summer, particularly in July. However, monthly data also indicated region-specific variations in seasonal activity.

These seasonal patterns were generally consistent with findings from other regions in Republic of Korea. For example, studies in Chungcheong and Jeolla Provinces reported peak larval activity in autumn, nymphs in spring, and adults in summer [25]. Similarly, in Gyeongsang Province, larvae peaked in autumn, nymphs in spring, and adults in both spring and summer [26]. In the present study, larvae peaked in autumn, nymphs in spring, and adults in summer; however, monthly fluctuations were observed. These findings suggest that the relatively higher latitudes and cooler climatic conditions of GG and GW Provinces may influence tick phenology and survival. Therefore, inter-regional differences in seasonal distribution likely reflect climatic and ecological factors, such as temperature, humidity, vegetation type, and the availability of host animals, complicating direct comparisons. Longitudinal studies across diverse geographic regions are warranted to further elucidate the seasonal dynamics and ecological determinants of tick populations in Republic of Korea.

A total of 4316 ticks were analyzed and categorized into 400 pools, with *A. phagocytophilum* detected in five of these pools. Regional comparisons of tick distribution and *A. phagocytophilum* infection rates revealed that *A. phagocytophilum* positivity was observed in both GW and GG Provinces; however, the infection rate was approximately four times higher in GW Province. The highest infection rate was identified in male *I. nipponensis* and *Haemaphysalis* spp. larvae, particularly in areas proximal to cattle farms. Seasonal variation was also evident, with the peak infection rate occurring in April (MIR: 0.31%; August: 0.21%; October: 0.15%). This may be attributed to increased tick activity during the spring season, as elevated temperatures and favorable environmental conditions in April are known to enhance tick questing behavior and facilitate host–vector contact. Accordingly, heightened precaution is warranted during outdoor activities near cattle farms during this period.

In a previous study conducted in the Gyeongsang Provinces, the MIR of *A. phagocytophilum* was reported as 0.7% [26], which was significantly higher than the 0.16% observed in this study. However, the previous detection was limited to tick-specific variants identified in *Haemaphysalis* spp., whereas both tick-specific and human pathogenic variants were confirmed in this study, indicating a more significant public health concern.

Additionally, previous studies in Gyeongsang Province reported higher MIRs in *I. nipponensis* compared to *Haemaphysalis* spp. [26]. This finding is consistent with the results of the present study. However, the high MIR observed for *I. nipponensis* in this study may be overestimated due to the relatively small number of positive pools compared to the total pools tested, indicating the need for further sampling in future research.

The 95% CI for the MIR calculated in this study ranged from 0 to 10.1%, indicating considerable statistical uncertainty. This wide interval is likely attributable to the small number of positive pools (*n* = 5) and the variability in the number of ticks per pool. These results underscore the need for more extensive and standardized sampling methods in future studies to improve the accuracy of prevalence estimates. They also suggest the possibility of community-level transmission and may serve as a valuable foundation for strengthening surveillance systems for tick-borne diseases.

*A. phagocytophilum* exhibits genetic diversity associated with its pathogenicity in humans and can be broadly categorized into human pathogenic and non-pathogenic variants. These variants differ in their nucleotide sequences, particularly in the *sdhC* gene region. Human pathogenic variants are typically restricted in distribution and are found in humans, animals, and ticks, whereas non-pathogenic variants are more widely distributed and have been primarily detected in ticks and certain animal hosts, such as deer [27].

In Republic of Korea, *A. phagocytophilum* has thus far been reported to be transmitted to humans exclusively by *I. nipponensis* [28]. Nevertheless, its detection in both *I. nipponensis* and the predominant *Haemaphysalis* spp. in a previous study [26], as well as in the present investigation, suggests a potential vector role for *Haemaphysalis* spp., especially considering that some studies have reported its presence exclusively in *H. longicornis* [29]. This finding highlights the need for further research into the vector competence of *Haemaphysalis* spp. for *A. phagocytophilum* transmission. Although conclusive evidence of human transmission via *Haemaphysalis* spp. remains lacking, infected animals may serve as reservoirs, and transmission through blood exposure has been proposed [12]. These findings highlight the importance of continued molecular surveillance and vector competence studies involving *Haemaphysalis* spp.

Tramsovarial transmission has been documented for *Babesia* [30] and *Rickettsia* [31] species in certain tick vectors. This route involves the direct passage of pathogens from an infected female tick to her progeny via the ova, thereby enabling pathogen persistence within tick populations independently of vertebrate hosts. These mechanisms provide an ecological basis for the long-term maintenance of such pathogens in the environment and constitute a persistent transmission risk to both human and animal populations.

In contrast, the capacity for transovarial transmission of *A. phagocytophilum* remains under investigation. Nonetheless, prior research [26,32], along with the present study, has identified *A. phagocytophilum* DNA in *Haemaphysalis* larvae that had not yet fed, indicating that the possibility of vertical transmission cannot be excluded. These findings underscore the need for ongoing surveillance and targeted preventive strategies to reduce the burden of tick-borne diseases in livestock-associated settings.

Epidemiological data indicate that rural populations are at greater risk for *A. phagocytophilum* infection compared with urban populations, particularly among individuals with livestock exposure [33]. The higher detection rate of *A. phagocytophilum* observed in GW Province—an area with a higher proportion of rural settlements compared to GG Province—underscores the need for continued surveillance, especially given the limited availability of historical data for comparison.

This study is the first to investigate *Anaplasma* sp. infection in ticks collected from livestock adjacent environments in GG and GW Provinces. The findings are expected to support a more systematic understanding of pathogen distribution within these regions. Although the overall detection rate was low, the presence of pathogens in unfed larvae and tick species with zoonotic potential underscores the ongoing risk of transmission to both humans and animals. However, due to the use of primers targeting the 16S rRNA gene, sequences corresponding to Ap-ha and Ap-V1 could not be analyzed in this study. Future research should consider using multiple primer sets targeting different genes to allow for more comprehensive genetic characterization.

Although Republic of Korea does not share a direct land border with China, GG and GW Provinces border North Korea, which shares a border with China. This geographical connection, along with the movement of migratory birds and wildlife through the region, makes these provinces potentially vulnerable to the transboundary spread of ticks and tick-borne pathogens. Thus, the current findings may serve as valuable indirect indicators of the ecological status of ticks and associated pathogens in North Korea, where direct surveillance is not feasible. Continued surveillance of tick species composition and infection rate in GG and GW Provinces is, therefore, warranted. Such efforts are essential for reducing the burden of tick-borne diseases and for enhancing regional public health preparedness and response capacity.

## Figures and Tables

**Table 1:** Species and developmental stages identification of ticks collected from grasslands near livestock farms in Gyeonggi and Gangwon Provinces, Republic of Korea.

Tick Species	Stage	No. of Collected Ticks by Region (%)			*p*-Value ^2^
		Gyeonggi	Gangwon	Total	
*Haemaphysalis* spp. ^1^	Larva	753	1013	1766 (40.91)	-
*Haemaphysalis longicornis*	Nymph	1007	1415	2422	<0.001
	Adult male	7	7	14	
	Adult female	28	24	52	
	Subtotal	1042	1446	2488 (57.65)	
*Haemaphysalis* *f* *lava*	Nymph	9	39	48	<0.001
	Adult male	1	0	1	
	Adult female	2	2	4	
	Subtotal	12	41	53 (1.23)	
*Ixodes nipponensis*	Adult male	5	1	6	0.011
	Adult female	2	0	2	
	Subtotal	7	1	8 (0.19)	
*Amblyomma testudinarium*	Nymph	0	1	1 (0.02)	-
Total		1814	2502	4316	

^1^ *Haemaphysalis* spp. larvae were grouped as *Haemaphysalis* spp. due to the difficulty in morphological differentiation of larvae at the species level. ^2^ Significant correlation (*p* < 0.05).

**Table 2:** Monthly and regional distribution of *Anaplasma*-positive ticks by species and developmental stage.

Month	Locality	No. of Tested Ticks	No. of Tick Pools	Tick Species	Stage	No. of Positive Pools (MIR ^1^)	Total No. of Positive Pool (MIR ^2^)
4	Gyeonggi	365	46	*I. nipponensis*	Male	1 (0.27)	3 (0.32)
	Gangwon	579	68	*I. nipponensis*	Male	1 (0.17)	
				*H. longicornis*	Nymph	1 (0.17)	
5	Gyeonggi	282	33	-	-	-	-
	Gangwon	373	42	-	-	-	
6	Gyeonggi	220	28	-	-	-	-
	Gangwon	294	35	-	-	-	
7	Gyeonggi	165	33	-	-	-	-
	Gangwon	240	43	-	-	-	
8	Gyeonggi	205	15				1 (0.21)
	Gangwon	263	18	*Haemaphysalis* spp.	Larva	1 (0.38)	
9	Gyeonggi	252	8	-	-	-	-
	Gangwon	394	12	-	-	-	
10	Gyeonggi	325	8	-	-	-	1 (0.15)
	Gangwon	359	11	*Haemaphysalis* spp.	Larva	1 (0.28)	
Total	Gyeonggi	1814	171	-	-	1 (0.06)	5 (0.12)
	Gangwon	2502	229	-	-	4 (0.16)	

^1^ Estimated as minimum infection rate (MIR). Calculated as MIR = number of positive pools/number of ticks tested in the region of interest × 100. ^2^ Estimated as minimum infection rate (MIR). Calculated as MIR = total number of positive pools/number of ticks tested in the month of interest × 100.

**Table 3:** *Anaplasma* infection rate in ticks based on tick species, developmental stage, and region.

Tick Species	Stage	No. of Tested Ticks (Pool)	No. of Positive Tick Pools by Region (%)			MIR ^1^	*p*-Value ^2^
			Gyeonggi	Gangwon	Total		
*Haemaphysalis* spp.	Larva	1766 (53)	0/17	2/36 (5.56)	2/53 (3.77)	0.11	<0.001
*H. longicornis*	Nymph	2422 (249)	0/105	1/144 (0.69)	1/249 (0.40)	0.04	
	Adult male	14 (14)	0/7	0/7	0/14	0	
	Adult female	52 (52)	0/28	0/24	0/52	0	
	sub total	2488 (315)	0/140	1/175 (0.57)	1/315 (0.32)	0.04	
*H. flava*	Nymph	48 (18)	0/4	0/14	0/18	0	
	Adult male	1 (1)	0/1	0	0/1	0	
	Adult female	4 (4)	0/2	0/2	0/4	0	
	sub total	53 (23)	0/7	0/16	0/23	0	
*I. nipponensis*	Adult male	6 (6)	1/5 (20.00)	1/1 (100)	2/6 (33.33)	33.33	
	Adult female	2 (2)	0/2	0	0/2	0	
	sub total	8 (8)	1/7 (14.29)	1/1 (100)	2/8 (25.00)	25.00	
*A. testudinarium*	Nymph	1 (1)	0	0/1	0/1	0	
Total		4316 (400)	1/171 (0.58)	4/229 (1.75)	5/400 (1.25)	0.12	

^1^ Estimated as minimum infection rate (MIR). Calculated as MIR = number of positive pools/total number of ticks tested × 100. ^2^ Significant correlation (*p* < 0.05).

## Data Availability

Data supporting the conclusions of this article are included within the article. The newly generated sequences were submitted to the GenBank database under accession numbers PV577431–PV577435. The datasets used and/or analyzed during the present study are available from the corresponding author upon reasonable request.

## References

[1] Madison-Antenucci S., Kramer L.D., Gebhardt L.L., Kauffman E. (2020). Emerging tick-borne diseases. Clin. Microbiol. Rev..

[2] Rosenberg R., Lindsey N.P., Fischer M., Gregory C.J., Hinckley A.F., Mead P.S., Paz-Bailey G., Waterman S.H., Drexler N.A., Kersh G.J. (2018). Vital signs: Trends in reported vector-borne disease cases—United States and territories, 2004–2016. MMWR Morb. Mortal. Wkly. Rep..

[3] Battilani M., De Arcangeli S., Balboni A., Dondi F. (2017). Genetic diversity and molecular epidemiology of *Anaplasma*. Infect. Genet. Evol..

[4] Woldehiwet Z. (2010). The natural history of *Anaplasma phagocytophilum*. Vet. Parasitol..

[5] Dumler J.S., Choi K.S., Garcia-Garcia J.C., Barat N.S., Scorpio D.G., Garyu J.W., Grab D.J., Bakken J.S. (2005). Human granulocytic anaplasmosis and *Anaplasma phagocytophilum*. Emerg. Infect. Dis..

[6] Chen S.M., Dumler J.S., Bakken J.S., Walker D.H. (1994). Identification of a granulocytotropic *Ehrlichia* species as the etiologic agent of human disease. J. Clin. Microbiol..

[7] Petrovec M., Lotric Furlan S., Zupanc T.A., Strle F., Brouqui P., Roux V., Dumler J.S. (1997). Human disease in Europe caused by a granulocytic *Ehrlichia* species. J. Clin. Microbiol..

[8] Zhang L., Cui F., Wang L., Zhang L., Zhang J., Yang S., Han J. (2009). *Anaplasma phagocytophilum* and *Ehrlichia* chaffeensis in Yiyuan County, Shandong. Infect. Dis. Inf..

[9] Ohashi N., Gaowa, Wuritu, Kawamori F., Wu D., Yoshikawa Y., Chiya S., Fukunaga K., Funato T., Shiojiri M. (2013). Human granulocytic anaplasmosis, Japan. Emerg. Infect. Dis..

[10] Heo E.J., Park J.H., Koo J.R., Park M.S., Park M.Y., Dumler J.S., Chae J.S. (2002). Serologic and molecular detection of *Ehrlichia chaffeensis* and *Anaplasma phagocytophila* (human granulocytic ehrlichiosis agent) in Korean patients. J. Clin. Microbiol..

[11] Lee S.J., Kim H.H., Kim J.Y., Yoo J.E., Gill B.C. (2021). Laboratory-based diagnostic test results for human granulocytic anaplasmosis in 2020. Public Health Wkly. Rep. PHWR.

[12] Dzięgiel B., Adaszek Ł., Winiarczyk S. (2016). Wild animals as reservoirs of *Anaplasma phagocytophilum* for humans. Prz. Epidemiol..

[13] Massung R.F., Slater K., Owens J.H., Nicholson W.L., Mather T.N., Solberg V.B., Olson J.G. (1998). Nested PCR assay for detection of granulocytic ehrlichiae. J. Clin. Microbiol..

[14] Massung R.F., Levin M.L., Munderloh U.G., Silverman D.J., Lynch M.J., Gaywee J.K., Kurtti T.J. (2007). Isolation and propagation of the Ap-Variant 1 strain of *Anaplasma phagocytophilum* in a tick cell line. J. Clin. Microbiol..

[15] Seo J.Y., Kim Y.J., Kim S.Y., Lee H.I. (2023). Molecular detection of *Anaplasma*, *Ehrlichia* and *Rickettsia* pathogens in ticks collected from humans in the Republic of Korea, 2021. Pathogens.

[16] Kim Y.-J., Seo J.Y., Kim S.Y., Lee H.I. (2022). Molecular Detection of *Anaplasma phagocytophilum* and *Ehrlichia* Species in Ticks Removed from Humans in the Republic of Korea. Microorganisms.

[17] Kim B., Lee Y.J., Kwak D., Seo M.G. (2024). Nationwide survey of vector-borne diseases in rodents and mites in Korea: *Anaplasma*, *Ehrlichia*, and *Rickettsia*. Animals.

[18] Kim M., Cho S., Park G., Kim J., Rieu M., Noh K.T., Ha S., Park Q., Kim D.H., Han S. (2024). Molecular detection of *Anplasma phagocytophilum*, *Borrelia theileri*, and Severe fever with thrombocytopenia syndrome virus (SFTSV) in military working dogs and ticks collected from the Republic of Korea Army garrisons in Gangwon Province in 2021–2022. Entomol. Res..

[19] Yamaguti N., Tipton V.J., Keegan H.L., Toshioka S. (1971). Ticks of Japan, Korea, and the Ryukyu islands. Brigh. Young Univ. Sci. Bull. Biol. Ser..

[20] Barlough J.E., Madigan J.E., Derock E., Bigornia L. (1996). Nested Polymerase Chain Reaction for Detection of Ehrlichia equi Genomic DNA in Horses and Ticks (Ixodes pacificus). Vet. Parasitol..

[21] Seo M.-G., Ouh I.-O., Lee H., Geraldino P.J.L., Rhee M.H., Kwon O.-D., Kwak D. (2018). Differential identification of *Anaplasma* in cattle and potential of cattle to serve as reservoirs of *Anaplasma capra*, an emerging tick-borne zoonotic pathogen. Vet. Microbiol..

[22] Tamura K., Stecher G., Kumar S. (2021). MEGA11: Molecular Evolutionary Genetics Analysis Version 11. Mol. Biol. Evol..

[23] Seo M.G., Noh B.E., Lee H.S., Kim T.K., Song B.G., Lee H.I. (2021). Nationwide temporal and geographical distribution of tick populations and phylogenetic analysis of severe fever with thrombocytopenia syndrome virus in ticks in Korea, 2020. Microorganisms.

[24] St. John H.K., Masuoka P., Jiang J., Takhampunya R., Klein T.A., Kim H.C., Chong S.T., Song J.W., Kim Y.J., Farris C.M. (2021). Geographic distribution and modeling of ticks in the Republic of Korea and the application of tick models towards understanding the distribution of associated pathogenic agents. Ticks Tick Borne Dis..

[25] Alkathiri B., Ahn K., Lee H., Cho Y.S., Youn S.Y., Seo M.G., Kwak D., Shin S., Lee S.H. (2023). Molecular epidemiology of *Theileria* species in ticks and its potential threat to livestock in the Republic of Korea. Acta Trop..

[26] Seo M.G., Lee H., Alkathiri B., Ahn K., Lee S.H., Shin S., Bae S., Kim K.T., Jang M., Lee S.K. (2023). Tick populations and molecular analysis of *Anaplasma* species in ticks from the Republic of Korea. Microorganisms.

[27] Liveris D., Aguero-Rosenfeld M.E., Daniels T.J., Karpathy S., Paddock C., Adish S., Keesing F., Ostfeld R.S., Wormser G.P., Schwartz I. (2021). A new genetic approach to distinguish strains of *Anaplasma phagocytophilum* that appear not to cause human disease. Ticks Tick Borne Dis..

[28] Lee S.H., Shin N.R., Kim C.M., Park S., Yun N.R., Kim D.M., Jung D.S. (2020). First identification of *Anaplasma phagocytophilum* in both a biting tick *Ixodes nipponensis* and a patient in Korea: A case report. BMC Infect. Dis..

[29] Oh J.Y., Moon B.C., Bae B.K., Shin E.H., Ko Y.H., Kim Y.J., Park J.H., Park B.K., Yoo J.Y., Chae J.S. (2009). Genetic identification and phylogenetic analysis of *Anaplasma* and *Ehrlichia* species in *Haemaphysalis longicornis* collected from Jeju Island, Korea. J. Bacteriol. Virol..

[30] Howell J.M., Ueti M.W., Palmer G.H., Scoles G.A., Knowles D.P. (2007). Transovarial transmission efficiency of *Babesia bovis* tick stages acquired by *Rhipicephalus* (*Boophilus*) *microplus* during acute infection. J. Clin. Microbiol..

[31] Moore T.C., Pulscher L.A., Caddell L., von Fricken M.E., Anderson B.D., Gonchigoo B., Gray G.C. (2018). Evidence for transovarial transmission of tick-borne rickettsiae circulating in Northern Mongolia. PLoS Negl. Trop. Dis..

[32] Ahn K., Alkathiri B., Lee S.H., Lee H., Kwak D., Cho Y.S., Lee H.S., Youn S., Yoo M.S., Kim J. (2025). Molecular detection of *Anaplasma phagocytophilum* in field-collected *Haemaphysalis* larvae in the Republic of Korea. Parasites Vectors.

[33] Zhang L., Liu H., Xu B., Zhang Z., Jin Y., Li W., Lu Q., Li L., Chang L., Zhang X. (2014). Rural residents in China are at increased risk of exposure to tick-borne pathogens *Anaplasma phagocytophilum* and *Ehrlichia chaffeensis*. Biomed. Res. Int..

